# Hydrogen peroxide signaling integrates with phytohormones during the germination of magnetoprimed tomato seeds

**DOI:** 10.1038/s41598-019-45102-5

**Published:** 2019-06-19

**Authors:** Anjali Anand, Archana Kumari, Meenakshi Thakur, Archana Koul

**Affiliations:** 1 Division of Plant Physiology, ICAR-Indian Agricultural Research Institute, New Delhi, 110 012 India; 2 Present Address: Central Insecticide Board and Registration Committee, Directorate of Plant Protection, Quarantine & Storage, Faridabad, Haryana 121 001 India; 3 Present Address: College of Horticulture and Forestry, Neri, Hamirpur, Dr. Y.S. Parmar University of Horticulture and Forestry, Nauni, Solan, Himachal Pradesh 173 230 India; 4 Present Address: Department of Plant Agriculture, University of Guelph, Ontario, N1G 2W1 Canada

**Keywords:** Molecular biology, Molecular biology, Plant sciences, Plant sciences

## Abstract

Seeds of tomato were magnetoprimed at 100 mT for 30 min followed by imbibition for 12 and 24 h, respectively, at 20 °C, to examine the biochemical and molecular changes involved in homeostasis of hydrogen peroxide (H_2_O_2_) and its signaling associated with hormone interactions for promoting vigor. The relative transcript profiles of genes involved in the synthesis of H_2_O_2_ like Cu-amine oxidase *(AO)*, receptor for activated C kinase 1 (RACK1) homologue (*ArcA2*) and superoxide dismutase *(SOD1 and SOD9)* increased in magnetoprimed tomato seeds as compared to unprimed ones with a major contribution (21.7-fold) from Cu-amine oxidase. Amongst the genes involved in the scavenging of H_2_O_2_ i.e, metallothionein (*MT1*, *MT3* and *MT4*), catalase (*CAT1*) and ascorbate peroxidase (*APX1* and *APX2*), *MT1 and MT4* exhibited 14.4- and 15.4-fold increase respectively, in the transcript abundance, in primed seeds compared to the control. We report in our study that metallothionein and RACK1 play a vital role in the reactive oxygen species mediated signal transduction pathway to enhance the speed of germination in magnetoprimed tomato seeds. Increased enzymatic activities of catalase and ascorbate peroxidase were observed at 12 h of imbibition in the magnetoprimed seeds indicating their roles in maintaining H_2_O_2_ levels in the primed seeds. The upregulation of *ABA 8*′*-hydroxylase* and *GA3 oxidase1* genes eventually, lead to the decreased abscisic acid/gibberellic acid (ABA/GA_3_) ratio in the primed seeds, suggesting the key role of H_2_O_2_ in enhancing the germination capacity of magnetoprimed tomato seeds.

## Introduction

Maintaining high standards of seed quality is of predominant significance for the establishment of a uniform crop stand and in turn profitable crop production. Seed priming techniques offer a well established solution for minimizing the spread of seedling emergence over time, so that the population of plants achieves similar size in order to produce marketable yield at the desired time of mechanical or manual harvest. Benefits of wet priming treatments have been realized in a number of horticultural crops where controlled seed hydration triggers the metabolic events that are usually activated during the germination process^1^. Magnetopriming presents similar advantage as the conventional hydro-, halo-, osmo-priming treatments *sans* the hydration-dehydration step that makes storage of primed seeds unfavourable. Seed vigour improvement by static and alternating magnetic fields have been reported in a number of horticultural crops^2–7^.

Tomato (*Solanum lycopersicum* L.) is an important commercial vegetable world over that serves the table purpose and processing. It is a rich source of energy, carotenoids, flavonoids, phenolics, mineral nutrients, vitamin C and dietary fibers, which are beneficial and serve as protective ingredients for human health^8^. However, various challenges like reducing land, declining natural resources and increasing biotic and abiotic stress affect the quality and production of this crop. In the quest for new technologies, magnetopriming has emerged as a viable approach for improving the seedling vigour that helps in establishing an adequate and uniform plant stand^7^. Magnetopriming induced seedling growth is accomplished by the production of reactive oxygen species (ROS) in the germinating soybean seed^9^. Reactive oxygen species like superoxide (O_2_^•−^) and hydrogen peroxide (H_2_O_2_) act as signaling molecules during the relief from dormancy and further progress towards germination. ROS may change the redox status of the seed and interact with germination related hormones to regulate the expression of genes that initiate the metabolic events responsible for germination^10,11^. Germination is controlled by two phytohormones that elicit an antagonistic effect on the germination process. Gibberellic acid positively regulates the germination process, whereas abscisic acid is involved in the induction and maintenance of seed dormancy^12^.

ROS concentration within the seed is tightly regulated by a multifactorial network of ROS removing enzymes such as superoxide dismutase, catalase and peroxidase that allow them to act as cellular messengers by existing at the critical levels, known as ‘oxidative window’. The advancement towards germination is not permitted in case the concentration of ROS is above or below the “oxidative window for germination”^10^. Seeds must be accorded with a ROS scavenging system that tightly regulates their concentration and enables ROS to act as cellular messenger. The seed antioxidant potential responsible for maintaining ROS levels during germination can be examined by observing the profiles of ROS scavengers in the germinating seeds. The present study was conducted to analyze the ROS network responsible for maintaining the level of H_2_O_2_ and its interaction with ABA-GA_3_ in germinating magnetoprimed tomato seeds.

## Results

### Speed of germination

**Figure 1:**
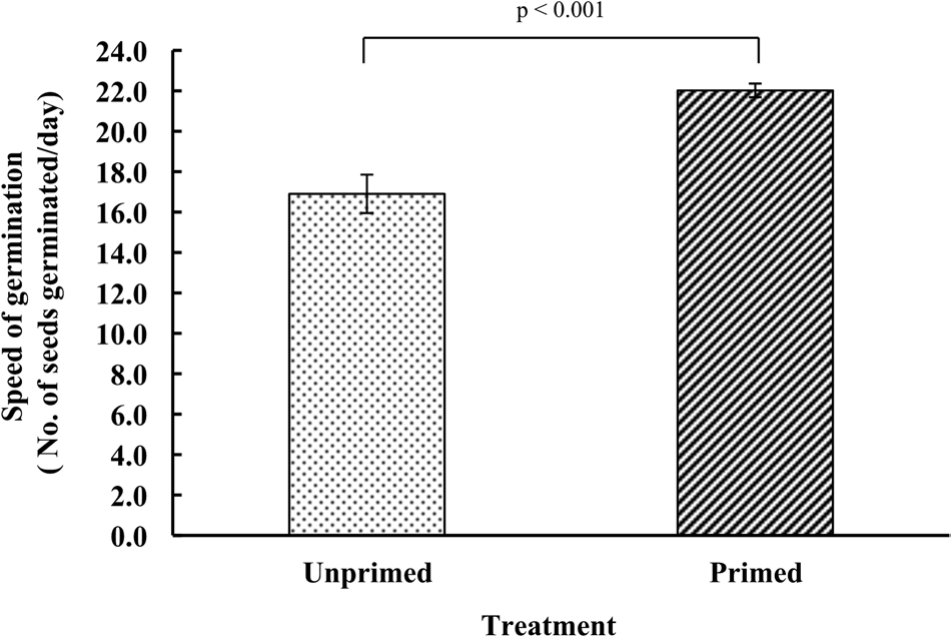
Effect of magnetic field exposure on speed of germination in primed and unprimed tomato seeds. Data represent mean ± S.E. of three biological replications replicated four times. Significant difference between treatments is indicated with the p value according to Student’s t test.

Speed of germination is an indicator for metabolic advancement of germination related events in the primed seeds. In magnetoprimed seeds, 22 seeds germinated per day and this rate of germination was 30.3% higher than unprimed ones (Fig. 1).

### Reactive oxygen species

**Figure 2:**
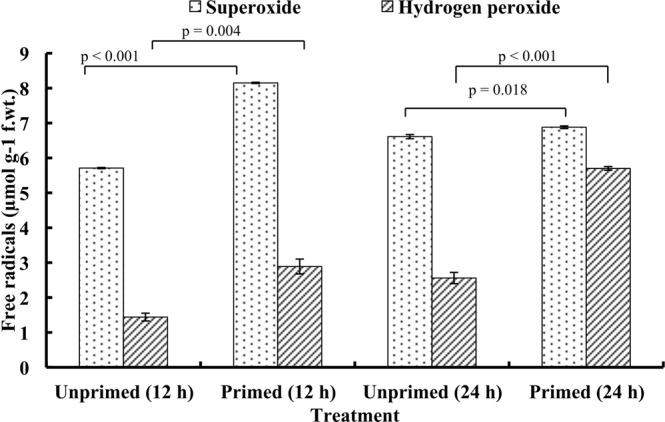
Effect of magnetic field exposure on generation of reactive oxygen species in germinating tomato seeds at different duration of imbibition. Data represent mean ± S.E. of three biological replications. Significant difference between treatments is indicated with the p value according to Student’s t test.

At 12 and 24 h of imbibition, primed seeds showed a significant increase in the superoxide radicals as compared to the unprimed seeds. At the cellular level, hydrogen peroxide content was 2-fold higher in the primed seeds as compared to the unprimed seeds at 12 and 24 h of imbibition respectively (Fig. 2).

### Localization of superoxide radical and hydrogen peroxide in seeds

**Figure 3:**
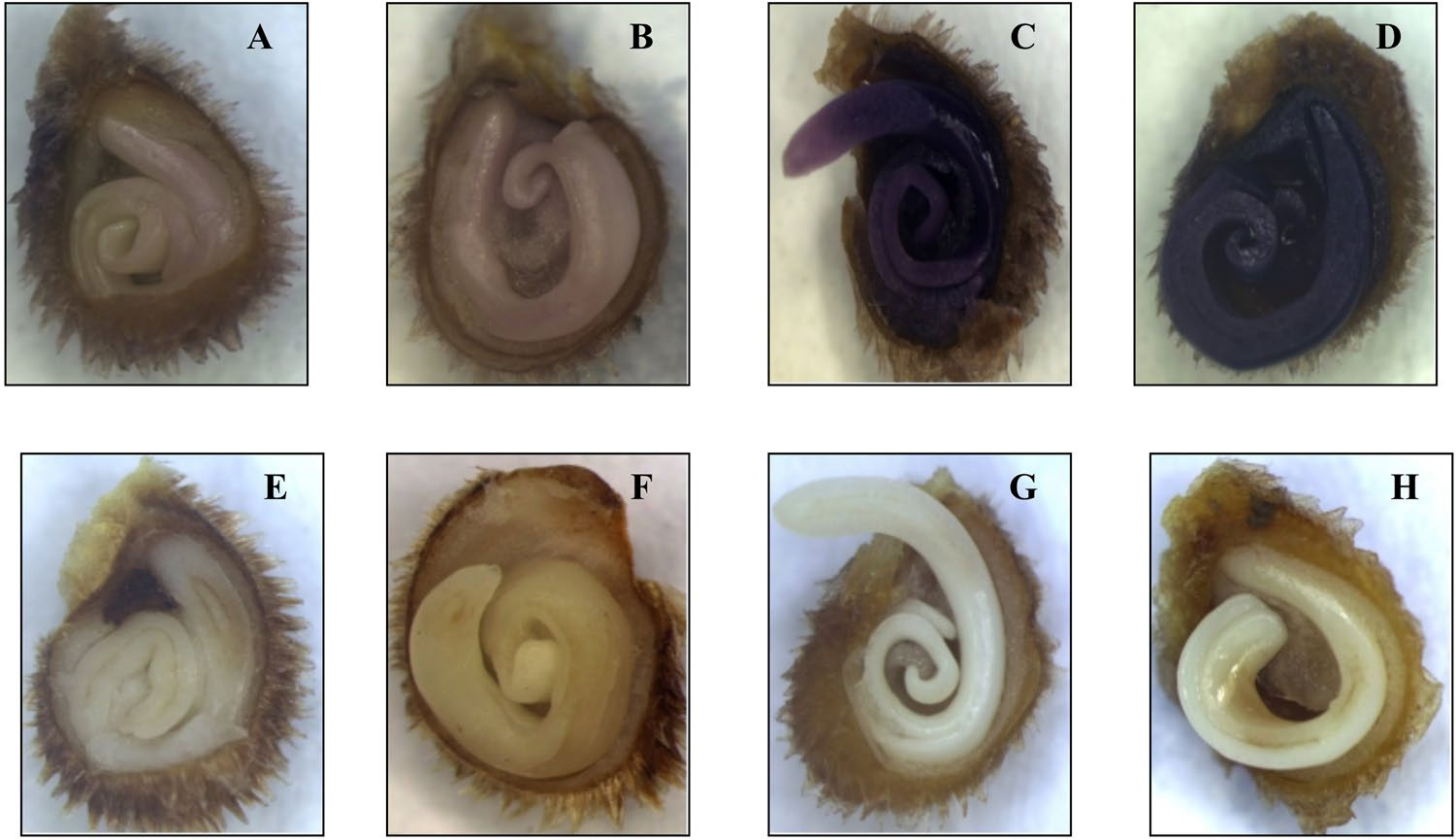
Photomicrographs showing localization of superoxide anion (NBT staining) and hydrogen peroxide (DAB staining) in tomato seeds. (**A**) superoxide anion in 12 h unprimed seeds; (**B**) superoxide anion in 12 h primed seeds; (**C**) superoxide anion in 24 h unprimed seeds; (**D**) superoxide anion in 24 h primed seeds. The formation of insoluble blue formazan precipitates in the endosperm of primed seeds indicate the strong accumulation of O_2_^•−^ as compared to unprimed seeds. (**E**) hydrogen peroxide in 12 h unprimed seeds; (**F**) hydrogen peroxide in 12 h primed seeds; (**G**) hydrogen peroxide in 24 h unprimed seeds; (**H**) hydrogen peroxide in 24 h primed seeds. Increased brown coloration in outer part of the endosperm of primed seeds shows higher production of H_2_O_2_ as compared to unprimed seeds.

At 12 and 24 h of imbibition, longitudinal sections of magnetoprimed tomato seeds showed insoluble blue formazan precipitates in the endosperm, indicating the strong O_2_^•−^ accumulation as compared to the unprimed seeds (Fig. 3A–D). Hydrogen peroxide detection by 3, 3′-diaminobenzidine (DAB) staining also showed an increase in brown color in the endosperm of primed seeds as compared to unprimed seeds. Unlike uniformly stained nitroblue tetrazolium (NBT) endosperm for O_2_^•−^ accumulation, H_2_O_2_ production was more on outer part of the endosperm in the primed seeds (Fig. 3E–H).

### Total antioxidant capacity

**Figure 4:**
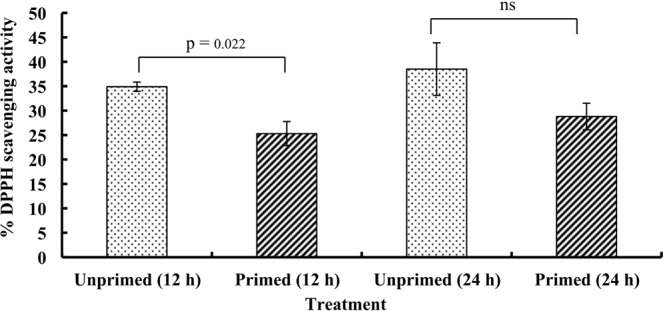
Effect of magnetic field exposure on total antioxidant capacity (% DPPH radical scavenging activity) in germinating tomato seeds at different duration of imbibition. Data represent mean ± S.E. of three biological replications. Significant difference between treatments is indicated with the p value according to Student’s t test. ns = non significant.

Radical scavenging assay using 2, 2-Diphenyl-1-picrylhydrazyl (DPPH) was conducted to measure the total antioxidant capacity that showed a significant decline in the primed seeds after 12 h of imbibition in comparison to that of the unprimed (Fig. 4).

### Antioxidant enzymes activities

**Figure 5:**
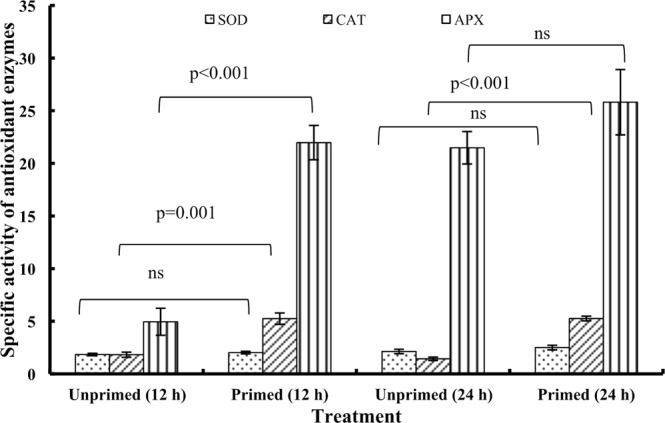
Effect of magnetic field exposure on antioxidant enzymes in germinating tomato seeds at different duration of imbibition [superoxide dismutase (units mg^−1^ protein), catalase (nmol H_2_O_2_ reduced min^−1^ mg^−1^ protein), ascorbate peroxidase (µmol ascorbate oxidized min^−1^ mg^−1^ protein)]. Data represent mean ± S.E. of four biological replications. Significant difference between treatments is indicated with the p value according to Student’s t test. ns = non significant.

Higher activities of antioxidant enzymes were observed in magnetoprimed seeds at 12 h as well as 24 h of imbibition (Fig. 5). Superoxide dismutase (SOD) catalyzes the dismutation of superoxide radical into either molecular oxygen or hydrogen peroxide. Magnetoprimed seeds exhibited a non-significant increase of 9.8 and 17.4% in SOD activity at 12 and 24 h of imbibition, respectively (Fig. 5). A noticeable 2.9- and 3.7-fold enhancement in catalase (CAT) activity was evident at 12 and 24 h of imbibition, respectively, in magnetoprimed seeds as compared to unprimed seeds. Ascorbate peroxidase (APX) activity in magnetoprimed seeds increased significantly by 4.4-fold at 12 h of imbibition compared to unprimed seeds. A non-significant increase of 20.2% was observed at 24 h in the primed seeds (Fig. 5).

### HPLC analysis of GA_3_ and ABA

At 12 h of imbibition, a significant increase of 70.2% in GA_3_ content was observed in the magnetoprimed seeds as compared to the unprimed seeds. The GA_3_ content decreased at 24 h of imbibition in both primed as well as unprimed seeds. Nonetheless, the GA_3_ content in the magnetoprimed seeds was 4.4-fold higher as compared to the unprimed seeds. In the magnetoprimed seeds, 18.3% decrease in ABA content was observed at 12 h of imbibition as compared to the unprimed seeds. The ABA content decreased at 24 h of imbibition, conversely, no change was observed in primed as well as unprimed seeds. At 12 and 24 h of imbibition, unprimed seeds exhibited high ABA/GA ratio as compared to primed seeds (Table 1).

**Table 1:** Effect of magnetic field exposure on plant hormones in germinating tomato seeds at different duration of imbibition.

Treatment	GA_3_ (µg g^−1^ fresh weight)	ABA (µg g^−1^ fresh weight)	ABA/GA_3_
Unprimed (12 h)	17.54 ± 3.75	0.19 ± 0.01	0.0108
Primed (12 h)	29.85* ± 1.75	0.16 ± 0.04	0.0050
Unprimed (24 h)	0.426 ± 0.15	0.04 × 10^−2^ ± 0.04 × 10^−4^	0.0009
Primed (24 h)	1.86* ± 0.08	0.04 × 10^−2^ ± 0.04 × 10^−4^	0.0002

Data represent mean ± S.E. of three biological replications. Asterisks indicate significant difference between primed and unprimed treatments at 12 and 24 h at p < 0.05, according to Student’s t test.

### Expression of genes involved in H_2_O_2_ synthesis, scavenging and signaling mechanism

**Figure 6:**
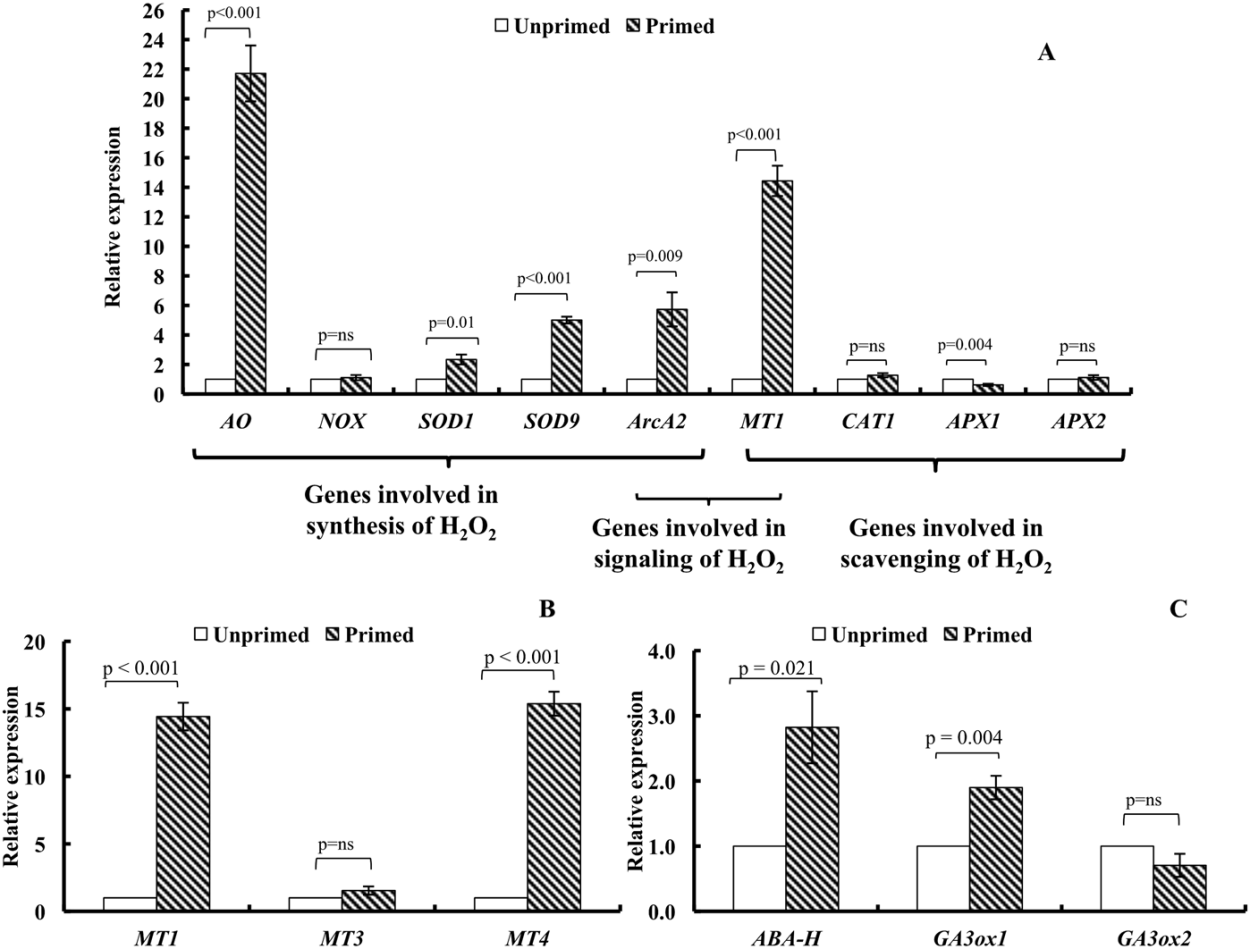
(**A**–**C**). Bar graphs depicting the effect of magnetic field treatment on the relative expression of various genes in germinating tomato seeds at 12 h of imbibition. (**A**) Relative expression of genes involved in the synthesis of H_2_O_2_, i.e. *AO* (Amine oxidase), *NOX* (NADPH oxidase), *SOD1* and *SOD9* (Superoxide dismutase 1 and 9), and *ArcA2* (receptor for activated C kinase 1/RACK1 homologue)^#^; scavenging of H_2_O_2_, i.e. *CAT1* (Catalase 1), *APX1* and *APX2* (Ascorbate peroxidase 1 and 2), *MT1* (Metallothionein 1); signaling of H_2_O_2_, i.e. *ArcA2* and *MT1*. (**B**) Relative expression of isoforms of Metallothionein (*MT1, MT3 and MT4*)^#^. (**C**) Relative expression of *ABA-H* (ABA 8′-hydroxylase) and *GA3ox1* and *GA3ox2* (Gibberellic acid 3 oxidase1 and 2). Data represent mean ± S.E. of three biological replicates with two technical replications. GAPDH gene was used as internal control to normalize the expression level. Fold change was calculated using 2^−ΔΔCT^ method that represents the changes in expression levels in the primed seeds as compared to the unprimed ones. Significant difference between treatments is indicated with the p value, according to Student’s t test. ns = non significant. qRT-PCR primers for all genes are provided in Table 2. ^**#**^*ArcA2* gene is involved in synthesis as well as signaling processes of H_2_O_2_, while *MT1* gene is involved in signaling as well as scavenging processes of H_2_O_2_.

Amongst the various genes involved in H_2_O_2_ synthesis, i.e. superoxide dismutase (*SOD1* and *SOD9*), amine oxidase (*AO*), NADPH oxidase (*NOX*) and RACK 1 homologue (*ArcA2*), the transcript levels of amine oxidase showed a significant increase (21.7-fold) in the magnetoprimed seeds over the unprimed ones at 12 h of imbibition. Transcript levels of *SOD1* and *SOD9* genes increased by 2.3- and 5-fold respectively, in the primed seeds as compared to the control. RACK 1 homologue (*ArcA2*) which is also involved in the signaling of H_2_O_2_ showed 5.7-fold increase in the primed seeds over the unprimed ones. Transcriptional analyses of genes involved in scavenging of H_2_O_2_, i.e., ascorbate peroxidase (*APX1* and *APX2)* and catalase (*CAT1*) showed non-significant increase in *APX2* and *CAT1* and significant downregulation of *APX1* in the magnetoprimed seeds over the unprimed ones (Fig. 6A). It is worth mentioning that the transcript abundance of metallothionein isoforms *MT1* and *MT4*, which are involved in scavenging and signaling function of H_2_O_2_ showed 14.4- and 15.4-fold increase respectively, in magnetoprimed seeds at 12 h of imbibition. However, *MT3* gene transcript showed 1.5-fold increase in magnetoprimed seeds (Fig. 6B). Genes involved in ABA deactivation, i.e. ABA 8′-hydroxylase (*ABA-H*) and GA synthesis, i.e. gibberellic acid 3 oxidase1 (*GA3ox1*) exhibited a 2.8- and 1.9- fold transcript abundance, respectively in the magnetoprimed seeds as compared to the unprimed ones at 12 h of imbibition (Fig. 6C).

## Discussion

Seed priming increases the seed performance by metabolically advancing the seed germination, resulting in the uniform seedling emergence. In our study, magnetopriming increased the speed of germination under the laboratory condition. At the biochemical level, magnetoprimed seeds exhibited significant increase in the production of reactive oxygen species, i.e. superoxide (O_2_^•−^) and hydrogen peroxide (H_2_O_2_) at 12 h of imbibition. Localization studies with NBT and DAB staining for superoxide and hydrogen peroxide, respectively, also confirmed the above findings. Many workers have reported the accumulation of ROS, i.e. hydrogen peroxide (H_2_O_2_)^6,13^, hydroxyl radicals (^•^OH)^14,15^ and superoxide radicals (O_2_^•−^)^14,16^ that enhanced dynamics of seed germination in various crop species. Our earlier studies on the magnetopriming mediated seed invigoration have also attributed the stimulation of seedling growth to the production of reactive oxygen species in the germinating seeds of soybean and tomato^7,9,17^. The probable sites of ROS generation are aerobic metabolism in mitochondria, peroxisomes or the apoplastic space in the germinating seeds, which can play a key signaling role in the achievement of major events occurring in the seed life, such as the germination or dormancy release. Among the various ROS produced, H_2_O_2_ is long lived and can diffuse across the membranes to reach targets far from their production sites. H_2_O_2_ is produced by numerous enzymes like oxalate oxidase, xanthine oxidase, membrane linked nicotinamide adenine dinucleotide phosphate oxidase (NADPH oxidase), amine oxidase and extracellular peroxidases, which can produce superoxide radicals that are subsequently converted to H_2_O_2_^18^. Significant reduction in total antioxidant capacity of the primed seeds at 12 h of imbibition also supports the accumulation of ROS in these seeds. Besides non-enzymatic antioxidant system (glutathione, ascorbic acid, α-tocopherol, carotenoids and phenolic compounds), the enzymatic defense system comprising of antioxidant enzymes like SOD, POD, CAT, GPX, GR, GST etc. is regarded to overcome the cascades of uncontrolled oxidation and protect the plant cells from ROS-induced oxidative damage^19,20^.

The role of magnetopriming in regulating enzymatic antioxidants has been well established by various researchers. SOD levels increased linearly with different times of imbibition (4 to 36 h) in the cherry tomato seedlings^7^. Increased antioxidative enzyme activities of SOD and CAT were observed in the magnetoprimed cucumber (*Cucumis sativus* L.) seeds^6^. In soybean, magnetic field increased the production of ROS, through the action of cell wall peroxidase^9^. In the present study, a non-significant increase in SOD activity was observed, although CAT and APX activities were significantly enhanced at 12 h of imbibition. ROS such as superoxide and H_2_O_2_ play a positive role in the germination and dormancy release by acting as secondary messengers in various developmental processes of the plants^21^. According to Barba-Espin *et al*.^22^ H_2_O_2_ acts as a ‘priming factor’ in pea seeds by altering the transcriptome, proteome and hormone levels, thus controlling the initial germination events in the seed. It is proposed to induce a mitogen activated protein kinase (MAPK) dependent decrease in the level of abscisic acid that is accompanied with carbonylation of seed storage proteins. However, a very precise regulatory mechanism of H_2_O_2_ accumulation by the antioxidant machinery of cell is crucial to achieve a perfect balance between the oxidative signaling that promotes germination; and the oxidative damage that prevents/delays germination^23^.

We examined the transcript abundance of some genes related to synthesis, scavenging and signaling of H_2_O_2_ to delve into the candidate genes responsible for H_2_O_2_ mediated invigoration by magnetopriming. Relative expression of genes involved in hydrogen peroxide production *viz*., amine oxidase (*AO*), superoxide dismutase (*SOD1* and *SOD9*) and RACK 1 homologue (*ArcA2*) increased significantly in the treated seeds. Amongst all the above genes, amine oxidase plays a major role in the production of H_2_O_2_. Increase in catalase and ascorbate peroxidase activities could not be related to the corresponding increase in the transcript levels of their respective genes. H_2_O_2_ regulates the expression of various genes involved in the positive control of germination, through protein carbonylation, activation, and modulation of kinase transduction cascades along with changes in the cellular redox states^24^. Bailly *et al*.^10^ reported higher accumulation of H_2_O_2_ in non-dormant imbibed seeds than in the dormant imbibed seeds and presented the concept of “oxidative window”, where critical range of ROS is responsible in promoting the germination process. Level of ROS above and below this range can impair the seed germination.

Besides the antioxidant enzymes, another candidate involved as an H_2_O_2_ scavenger and signaling is metallothionein^25,26^. Transgenic *Arabidopsis* seeds overexpressing the metallothionein genes obtained from sacred lotus (*Nelumbo nucifera* Gaertn.) and rice exhibited improved resistance against accelerated ageing^27^ and salt stress^28^. The *MT* gene (encoding a type 2 Metallothionein or *MT1*) induced by oxidative stress, was up-regulated during *Silene* species seed rehydration^29^. Magnetic stimulation of tomato seeds resulted in 15.4- and 14.4-fold increase in the transcript level of metallothionein genes *MT4* and *MT1*, respectively in magnetoprimed seeds suggesting the role of metallothionein during ROS signaling in germination of magnetoprimed seeds. To the best of our knowledge, this is the first report on the involvement of metallothionein genes in the signaling pathway mediated by ROS post magnetopriming, although similar reports are available, where ROS generated during non-thermal microwave electromagnetic fields activate metalloproteases, which stimulate epidermal growth factor receptor (EGFR) followed by the latter activating the ERK (extracellular-signal-regulated kinase) MAPKs cascade^30^. Besides MAPK signaling pathway that coordinates the regulation of seed germination^23^ at transcriptome and proteome levels, receptor for activated C kinase1 (RACK1), a member of the tryptophan-aspartate repeat family of proteins, is also involved in hydrogen peroxide signaling and ABA catabolism during the germination^31^ process. In our experimental data, upregulation of RACK1 gene (*ArcA2*) suggests its participation in signal transduction pathway as reported by Chang *et al*.^32^, where RACK1 interacts with serine/threonine protein kinase C and plays a key role in the signal transduction process. Further studies can provide the evidence for elucidating the role of metallothionein in the scavenging or signaling pathway.

Hydrogen peroxide acts as a signaling hub for the regulation of both seed dormancy as well as germination by concomitant trigger of ABA catabolism and GA_3_ biosynthesis. The antagonism between GA_3_ and ABA plays a key role in controlling the seed germination^33–36^. For example, GA_3_ induces transcription of α-amylase in the aleurone layer of cereal seeds that is significantly suppressed by ABA^37^. In case of high concentration of ABA, the expression of GA_3_ biosynthesis genes is inhibited, so that equilibrium of these two hormones jointly controls seed dormancy and germination^38^. There is lot of information on cross talk between ROS and phytohormone signaling for dormancy release^39–41^. In *Arabidopsis*, H_2_O_2_ can promote the germination by enhancement of ABA catabolism and GA_3_ biosynthesis. In magnetoprimed seeds, reduction in ABA content and increase in GA_3_ content was observed suggesting the active role of magnetopriming technique in dormancy release and seed germination. The concentration of GA_3_ decreased at 24 h, although the trend remained similar. The concentration of ABA also decreased at 24 h, but no difference was observed between the primed and unprimed ones. At 12 and 24 h of imbibition, unprimed seeds exhibited high ABA/GA_3_ ratio as compared to the primed ones, suggesting the increased dormancy release in the primed tomato seeds. The ratio of ABA and GA_3_ is related to the seed dormancy and seed germination. At the molecular level, the ABA/GA_3_ balance is partly determined by the strong antagonistic control of ABA and GA_3_ on each other by inversely regulating the transcription of their metabolic genes^36^.

**Figure 7:**
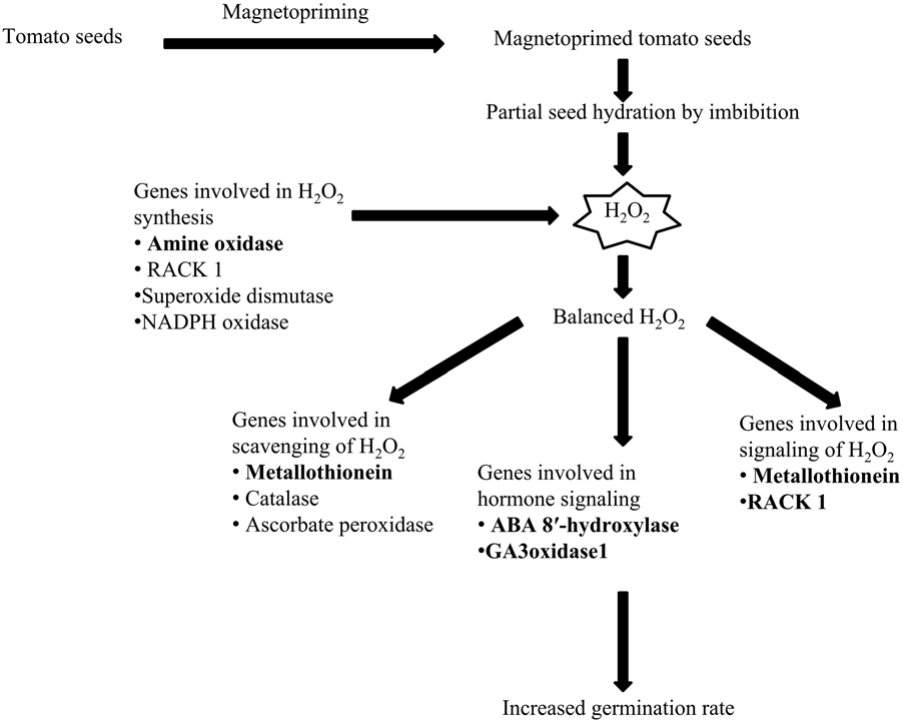
Schematic model of regulation of H_2_O_2_ for oxidative signaling in magnetoprimed tomato seeds. Bold letters show major contribution of genes involved in the metabolic process. Primimg of tomato seeds with magnetic field of 100 mT (30 min) followed by imbibition for 12 h led to increased production of hydrogen peroxide in the primed seeds. Amongst the genes involved in H_2_O_2_ production (amine oxidase, NADPH oxidase and superoxide dismutase), amine oxidase is the key player for H_2_O_2_ synthesis. Metallothionein and receptor for activated C kinase 1 are proposed to be responsible for signaling process, besides scavenging and production of H_2_O_2,_ respectively. Thus, H_2_O_2_ acts as pivotal signaling molecule by integrating with phytohormones through reduction in ABA and increase in GA levels for increasing the speed of germination in magnetoprimed tomato seeds.

Relative gene expression of ABA catabolism and GA_3_ synthesis genes, showed 2.8- and 1.9-fold increase in *ABA 8*′*-hydroxylase* (*ABA-H/CYP707A2*) and *GA3oxidase1* (*GA3ox1*), respectively. Increased expression of genes involved in ABA deactivation and GA synthesis suggested that this regulation of induction during magnetopriming may occur through ABA-GA_3_ crosstalk. H_2_O_2_ enhances GA biosynthesis by increasing the expression of GA biosynthetic pathway genes such as *GA3ox* and *GA20ox* genes^38^. Various reports corroborate these findings where exogenous hydrogen peroxide enhanced the expression of *CYP707A* genes that encode ABA 8′-hydroxylases and GA_3_ synthesis in dormant *Arabidopsis* seeds^38^. The modulation in hormonal balance due to inhibition of ABA and activation of GA_3_ signaling explained the close knit interaction with H_2_O_2_ in regulating vigour in the magnetoprimed seeds. However, in sunflower plants, El-Maarouf-Bouteau *et al*.^42^ opined that ROS operates along with ABA at transcriptional level by reducing the number of key transcripts rather than stimulating GA or ethylene signaling pathways. They attributed ROS action to the series of genes involved in calcium and redox signaling. Barba-Espin *et al*.^43^ proposed the direct role of H_2_O_2_ as a signaling molecule within the phytohormone network that  lead to the induction of proteins that are associated with the plant signaling and development processes during the pea seed germination. Thus, H_2_O_2_ acts as a hub that balances and integrates phytohormone interactions for enhancing the germination and vigour in the magnetoprimed tomato seeds. We have postulated a schematic diagram to depict the key players in H_2_O_2_ mediated signaling for seed invigoration in the magnetoprimed tomato seeds (Fig. 7).

In conclusion, our results demonstrate that balanced hydrogen peroxide through concerted action of synthesis and scavenging system, act as a signaling centre for the regulation of improved vigor by triggering both ABA catabolism and GA_3_ biosynthesis. Besides, metallothionein plays a pivotal role in the scavenging and oxidative signaling processes by means of hydrogen peroxide.

## Materials and Methods

Tomato seeds var. Pusa Rohini were exposed to the static magnetic field strength of 100 mT for 30 mins. Speed of germination was determined in both primed and unprimed seeds. Seeds were further imbibed for 12 and 24 h, respectively at 20 °C for the determination of free radicals, total antioxidant capacity, antioxidative enzymes and hormones (ABA and GA_3_). Transcript profiling of genes involved in H_2_O_2_ production, scavenging and signaling in the tomato seeds imbibed for 12 h after magnetopriming was also performed.

### Speed of germination

Hundred primed and unprimed seeds were kept in four replications of 25 seeds each on wet filter paper in Petri-dish. The seeds were incubated at 20 °C and a daily count of germination was taken until no more seeds germinated. The speed of germination (Σn/t) was calculated following the formula given by Maguire^44^ and expressed as number of seeds germinated/day.

$$\sum n/t=\frac{{\rm{Number}}\,{\rm{of}}\,{\rm{seeds}}\,{\rm{germinated}}}{{\rm{Day}}\,{\rm{of}}\,{\rm{the}}\,{\rm{first}}\,{\rm{count}}}+----+\frac{{\rm{Number}}\,{\rm{of}}\,{\rm{seeds}}\,{\rm{germinated}}}{{\rm{Day}}\,{\rm{of}}\,{\rm{the}}\,{\rm{final}}\,{\rm{count}}}$$

where, n = No. of seeds germinated and t = Time of count.

### Measurement of reactive oxygen species

#### Determination of superoxide (O_2_^•−^) radical

Quantification of superoxide free radical was done by measuring its capacity to reduce nitroblue tetrazolium chloride (NBT) in primed and unprimed seeds according to the method of Chaitanya and Naithani^45^. Homogenization of tomato seeds (0.5 g) was performed in pre-cooled phosphate buffer (0.2 M, pH 7.2) followed by centrifugation at 10,000 x g for 10 min at 4 °C. A 3 ml reaction mixture was prepared containing 100 µl supernatant in 0.75 mM NBT, 25 mM sodium carbonate, 0.1 mM EDTA and 13.3 mM L-methionine followed by incubation at 30 °C in a water bath for 10 min. The absorbance was recorded spectrophotometrically at 540 nm. The content of superoxide anion was estimated using a molar absorption coefficient of 12.8 mM^−1^ cm^−1^ and expressed as µmol g^−1^ fresh weight.

#### Hydrogen peroxide measurement (H_2_O_2_)

Hydrogen peroxide content was determined by the formation of titanium-hydro peroxide complex^46^. Tomato seeds (0.5 g) were homogenized in chilled acetone and the homogenate was filtered using Whatman No. 1 filter paper. To the filtrate, 2 ml of titanium reagent and 2.5 ml of ammonium hydroxide solution were added in order to precipitate the titanium-hydro peroxide complex. The total reaction mixture was centrifuged at 10,000 × g for 10 min and the precipitate was further dissolved in 5 ml of 2 M concentrated sulphuric acid and then re-centrifuged. The absorbance of the supernatant was read at 415 nm against blank and H_2_O_2_ content was expressed as μmol g^−1^ fresh weight.

#### Localization of superoxide radical and hydrogen peroxide in seeds

To examine the localization of superoxide anion (O_2_^•−^) and hydrogen peroxide (H_2_O_2_), seeds were imbibed with distilled water for 12 and 24 h followed by incubation of hand-cut longitudinal sections in 6 mM nitroblue tetrazolium (NBT) or 4.7 mM 3, 3′-diaminobenzidine (DAB) in 10 mM Tris HCl buffer (pH 7.4) for 30 min for superoxide and hydrogen peroxide, respectively^47^. The superoxide anion and hydrogen peroxide were seen as areas of dark-blue and brown coloration under a stereomicroscope (Leica M20F5, Germany), respectively.

#### Total antioxidant activity

Radical scavenging assay with 2, 2-Diphenyl-1-picrylhydrazyl (DPPH) was used to measure the total antioxidant activity in seeds imbibed for 12 and 24 h following the procedure of Brand and Williams^48^. Primed and unprimed seeds (0.2 g) were extracted in 1 ml methanol and 100 µl of methanolic seed extract was added to 3.9 ml of 0.06 mM DPPH solution in methanol. The solution was incubated for 30 min in dark at room temperature followed by recording the absorbance at 515 nm. The percentage of DPPH radical scavenging activity was calculated using the formula:

$${\rm{ \% }}\,{\rm{D}}{\rm{P}}{\rm{P}}{\rm{H}}\,{\rm{r}}{\rm{a}}{\rm{d}}{\rm{i}}{\rm{c}}{\rm{a}}{\rm{l}}\,{\rm{s}}{\rm{c}}{\rm{a}}{\rm{v}}{\rm{e}}{\rm{n}}{\rm{g}}{\rm{i}}{\rm{n}}{\rm{g}}\,{\rm{a}}{\rm{c}}{\rm{t}}{\rm{i}}{\rm{v}}{\rm{i}}{\rm{t}}{\rm{y}}=1-\frac{{\rm{A}}{\rm{b}}{\rm{s}}{\rm{o}}{\rm{r}}{\rm{b}}{\rm{a}}{\rm{n}}{\rm{c}}{\rm{e}}\,{\rm{o}}{\rm{f}}\,{\rm{s}}{\rm{a}}{\rm{m}}{\rm{p}}{\rm{l}}{\rm{e}}}{{\rm{A}}{\rm{b}}{\rm{s}}{\rm{o}}{\rm{r}}{\rm{b}}{\rm{a}}{\rm{n}}{\rm{c}}{\rm{e}}\,{\rm{o}}{\rm{f}}\,{\rm{c}}{\rm{o}}{\rm{n}}{\rm{t}}{\rm{r}}{\rm{o}}{\rm{l}}({\rm{D}}{\rm{P}}{\rm{P}}{\rm{H}})}\times 100$$

#### Antioxidant enzyme extraction and their assays

Germinating seed samples (1 g) at 12 and 24 h of imbibition were homogenized using 50 mM potassium phosphate buffer (pH 7.0) containing 1 mM EDTA and 1% (w/v) polyvinyl pyrrolidone along with 0.2 mM ascorbate for the ascorbate peroxidase assay to protect APX activity. The homogenate was further centrifuged at 10,000 × g for 30 min at 4 °C and the supernatant was used for the following assays:

#### Superoxide dismutase (SOD) activity

SOD activity was measured by calculating the amount of enzyme induced 50% decrease in the absorbance of formazone by nitroblue tetrazolium (NBT) in comparison with the tubes lacking the enzyme^49^. The absorbance was recorded at 560 nm and the specific activity was described as units mg^−1^ protein.

#### Catalase (CAT) activity

CAT activity was estimated by observing the disappearance of H_2_O_2_ (Ɛ = 39.4 mM^−1^ cm^−1^)^50^. A 3 ml reaction mixture was prepared which contained sodium phosphate buffer (50 mM, pH 7.0) and 10 mM H_2_O_2_. Absorbance was measured at 240 nm and the specific activity was expressed as nmol H_2_O_2_ reduced min^−1^ mg^−1^ protein.

#### Ascorbate peroxidase (APX) activity

APX activity was measured by measuring the decrease in absorbance at 290 nm (Ɛ = 2.8 mM^−1^ cm^−1^) for 1 min in 3 ml reaction mixture containing 50 mM potassium phosphate buffer (pH 7.0), 0.1 mM EDTA, 0.5 mM ascorbic acid, 1.5 mM H_2_O_2_ and 0.1 ml enzyme extract. The specific activity was expressed as µmol ascorbate oxidized min^−1^ mg^−1^ protein^51^.

#### Determination of GA_3_ and ABA

Imbibed tomato seeds were homogenized using phosphate buffer (0.05 M, pH 7.5) (1:5 w/v) containing 0.2% sodium diethyldithiocarbamate according to the modified protocol of Wurst *et al*.^52^. The homogenate was kept overnight at 4 °C in a shaker (150 rpm) followed by centrifugation at 10,000 rpm at 4 °C for 10 min. The supernatant was partitioned in a separating funnel using diethyl ether. The aqueous layer was collected and its pH was adjusted to 2.5 using 1 N HCl followed by partitioning twice with petroleum ether. Aqueous layer was again collected and partitioned thrice using diethyl ether. The ether phase was filtered over sodium sulphate crystals using Whatman No. 1 filter paper which was followed by the lyophilization step. The lyophilized sample was dissolved in acetonitrile:water (26:74) and analyzed in Agilent zorbax eclipse XDB C-18 column (4.6 mm × 250 mm Agilent Technologies 1100/1200 Series, USA) maintained at 35 °C with the UV-Visible detector with a flow rate of 0.8 ml/min. The standards were also included with each group of samples loaded onto the HPLC at the same time as a control on the detector response.

#### RNA extraction and cDNA synthesis

Total RNA was extracted from 0.5 g seeds using Trizol RNA isolation reagent (Invitrogen, Carlsbad, CA, USA). Single-stranded cDNAs were synthesized from 1 µg of RNA using a cDNA synthesis kit according to the manufacturer’s instructions (Takara Clontech, Japan).

#### Gene expression study

Quantitative Real Time PCR was performed with a gene specific primer pair and Glyceraldehyde 3-phosphate dehydrogenase (GAPDH) primer pair as an internal control. Primer sequences (Table 2) were designed on the exon-exon boundaries using the Primer Quest Tool of Integerated DNA technologies (IDT Inc., Belgium) and were synthesized by Sigma-Aldrich, India. Reactions were performed on a Biorad system (USA) using SYBR-Green Real-time PCR Master Mix (Takara, Japan) according to the manufacturer’s instructions. The thermocycling parameters were; hot start at 95 °C for 3 min, 39 cycles at 95 °C for 10 sec and 60 °C for 30 sec. The specificity of the PCR reactions was assessed after the amplification by the presence of a single peak in the dissociation curve and by the estimation of the size of the amplified product on 2.5% agarose gel. The comparative cycle threshold (CT) method, i.e., (2^−ΔΔCT^)^53^ was used to check the differences in relative gene expression levels between the primed and unprimed seeds.

**Table 2:** Primers used to check the expression of genes involved in hydrogen peroxide production, scavenging and signaling mechanisms.

S.No.	Enzyme/Property	Gene Name	Accession No.	Primer pair	
1.	Cu containing amine oxidase	AO	NM_001310065.1	F	ATACGGGTTCGGGTTACA
				R	CCATCAGCTGCCACAAATA
2.	NADPH oxidase	*NOX*/*RBOH1*	NM_001247197.2	F	GCCGAATTGGAGGAAAGTAT
				R	CAGAGTTGGCTGAGTTCTTT
3.	Superoxide dismutase Cu-Zn	*SOD1*	NM_001311084.1	*F*	CCACAAATGGCTGTATGTCAAC
				*R*	TACCAAGATCACCAGCATGAC
4.	Superoxide dismutase Mn	*SOD9*	NM_001330621	F	TTTAACGGCGGAGGTCATATT
				R	CAATAGCCCAGCCAAGAGAA
5.	Receptor for activated C kinase 1 (RACK 1) homologue	*ArcA2*	AB022687.1	F	GTGGCAAGGATGGAGTTATT
				R	AGGACTAAAGCAGAGAGTATGA
6.	Metallothionein type 2	*MT1*	NM_001247117.2	F	GTTGAAACAGAGCAGAGATCA
				R	TTCAACAGACAGACACACTAAA
7.	Metallothionein type 2	*MT3*	NM_001247125.2	F	GTGGATCTGGCTGCAAGT
				R	GTGCAACACCTTCAACGAT
8.	Metallothionein type 2	*MT4*	NM_001247362.2	F	TGGATCTGGCTGCAAGTG
				R	CTCAGCCACTCCATAGTTCTTC
9.	Catalase	*CAT1*	KU933832.1	F	GTACCCGATTCCTTCTTGTG
				R	TCCCATGATCTGTACCTCTC
10.	Ascorbate peroxidase	*APX1*	NM_001247853.2	F	ACGATGATATTGTGACACTCTTCCA
				R	AAGCGATGAAACCACAAAAACA
11.	Ascorbate peroxidase	*APX2*	NM_001247702.2	F	TGGGAGGGTGGTGACATATTTT
				R	TTGAAGTGCATAACTTCCCATCTTT
12.	ABA 8′- hydroxylase	*ABA-H/CYP707A2*	HQ008774.1	F	CCATCGCGATAACATCACTC
				R	TTCTTGAGCTCCTCTCTGTAT
13.	Gibberellic acid3 oxidase1	*GA3ox1*	AB010991.1	F	GATGTGCTGCCTTACAACTA
				R	GGTAGAATCCGTATGTGCTG
14.	Gibberellic acid3 oxidase2	*GA3ox2*	NM_001246926.3	F	CGGAGTCCATTCCAGTAATC
				R	GGTTAAGTAATCGGTGCGATA
15.	Glyceraldehyde 3-phosphate dehydrogenase	*GAPDH*	NM_001247874.2	F	GGAGACAATAGACCAAGCATATT
				R	TGAATAATCAAGTCCACCACTC

### Statistical analysis

Differences in the speed of germination, ROS, total antioxidant capacity, antioxidant enzymes and expression analysis of hydrogen peroxide related genes between primed and unprimed seeds were calculated using Student’s t-test at p < 0.05 of Excel software. Values for all parameters are presented as mean ± S.E.
